# Effects of twelve-week inspiratory muscle vs. plyometric training on pulmonary function and pupillary light reflex in youth middle-distance runners—a three-arm randomized controlled trial

**DOI:** 10.3389/fphys.2026.1781766

**Published:** 2026-10-14

**Authors:** Vinu W., Josyula Tejaswi, Dilshith Azeezul Kabeer, Debajit Karmakar, Ibnu Noufal Kambitta Valappil, Koulla Parpa, Borko Katanic, Vlad Adrian Geantă, Jolita Vveinhardt, Karuppasamy Govindasamy

**Affiliations:** 1 Department of Physical Education & Sports, Pondicherry University, Puducherry, India; 2 Symbiosis School of Sports Sciences, Symbiosis International (Deemed University), Pune, Maharashtra, India; 3 Department of Physical Education Pedagogy, Lakshmibai National Institute of Physical Education, Gwalior, India; 4 Faculty of Sport and Exercise Science, UCLan University of Cyprus, Pyla, Cyprus; 5 Montenergin Sports Academy, Podgorica, Montenegro; 6 Department of Physical Education and Sport, Faculty of Physical Education and Sport, Aurel Vlaicu University of Arad, Arad, Romania; 7 Institute of Sport Science and Innovations, Lithuanian Sports University, Kaunas, Lithuania; 8 Department of Sports, Recreation and Wellness, Symbiosis International (Deemed University), Hyderabad Campus, Modallaguda (V), Nandigama (M), Rangareddy, Telangana, India

**Keywords:** athletic performance, autonomic funct, middle-distance running, neuromuscular conditioning, pupillometry, respiratory training, spirometry, youth athletes

## Abstract

**Background:**

Inspiratory muscle training (IMT) and plyometric exercise are established conditioning modalities that elicit distinct physiological adaptations, yet their comparative effects on respiratory function and autonomic regulation in youth athletes remain unclear. The pupillary light reflex (PLR) provides a non-invasive index of autonomic activity and may offer new insight into training-induced adaptations. This study examined the effects of a 12-week IMT versus plyometric training program on pulmonary function and PLR dynamics in youth middle-distance runners.

**Methods:**

Ninety youth middle-distance runners (aged 14–18 years) were randomly assigned to an IMT group (n = 30), a plyometric training group (n = 30), or a control group (n = 30). The prespecified primary outcome was forced expiratory volume in 1 second (FEV_1_); secondary outcomes included other spirometry parameters (forced expiratory volume [FEV_1_], forced vital capacity [FVC], forced expiratory volume/forced vital capacity [FEV_1_/FVC] ratio, peak expiratory flow [PEF], and maximal inspiratory pressure [MIP]) and PLR metrics (initial diameter, constriction velocity, maximum constriction, re-dilation velocity) served as secondary/exploratory outcomes. Assessments were conducted at baseline and 12 weeks (no interim timepoint was assessed). Of 90 randomized participants, 86 (95.6%) completed the study and were included in the complete-case primary analysis.

**Results:**

IMT led to marked gains in FEV_1_ (4.12 ± 0.23 to 4.45 ± 0.25 L, p < 0.001), FVC (4.89 ± 0.31 to 5.28 ± 0.33 L, *p* < 0.001), and PEF (628 ± 45 to 672 ± 48 L/min, *p* < 0.001). PLR constriction velocity also improved significantly (5.2 ± 0.8 to 6.1 ± 0.9 mm/s, *p* < 0.01). In comparison, plyometric training yielded modest but significant improvements in FEV_1_ (4.08 ± 0.26 to 4.25 ± 0.28 L, *p* < 0.05) and increased PLR re-dilation velocity (3.1 ± 0.6 to 3.8 ± 0.7 mm/s, *p* < 0.05). No significant changes were observed in the control group.

**Conclusion:**

In this complete-case analysis, IMT produced greater adjusted improvements in pulmonary function than plyometric training in youth middle-distance runners, while both modalities were associated with distinct changes in pupillary light reflex indices (constriction velocity with IMT; re-dilation velocity with plyometric training) that may reflect differential autonomic adaptation. As running performance was not assessed, these findings should be interpreted as physiological rather than performance evidence, and the potential value of combining both modalities warrants prospective testing.

## 1 Introduction

The optimization of respiratory function and autonomic nervous system efficiency represents a critical component of athletic performance development, particularly in middle-distance running where aerobic capacity and neuromuscular coordination are paramount (Migliaccio et al., 2023). The interaction of these two systems plays a more central role in youth athletes, where motor control and energy metabolism develop rapidly during maturation. Youth athletes aged 14–18 years represent a unique population characterized by ongoing physiological maturation, making this developmental window particularly responsive to targeted training interventions (Jayanthi et al., 2022). Therefore, researchers have investigated training modalities that directly (breathing-based methods, such as inspiratory muscle training and breath control exercises) or indirectly affect the respiratory muscles (e.g., plyometric, resistance, or endurance training) to improve respiratory function (Hartz et al., 2018).

Inspiratory muscle training (IMT) has also emerged as a targeted intervention for the diaphragm and accessory respiratory muscles, utilizing principles of progressive overload (Shei et al., 2022). IMT may induce hypertrophy of the inspiratory muscles, thereby enhancing the inspiratory muscle pump’s capacity to regulate intrathoracic pressure (Zhang et al., 2020). It has been hypothesized that these adaptations could improve alveolar ventilation efficiency and increase parasympathetic activity within the autonomic nervous system by reducing respiratory-related afferent signalling (Illi et al., 2012), although this specific mechanism was not directly measured in the present study. Previous research has demonstrated that IMT can enhance inspiratory muscle strength, reduce perceived exertion during exercise, and improve exercise performance in adult populations (Illi et al., 2012; Manifield et al., 2021). However, the respiratory muscles, thoracic volume, and alveolar surface area, in proportion to the rib cage, continue to develop in individuals during adolescence (Archer et al., 2023). Furthermore, the balance of sympathetic and parasympathetic tone during adolescence may vary depending on circumstances (Harteveld et al., 2021). Therefore, autonomic responses to IMT may not be as stable as in adults. Finally, neuromuscular coordination development has been proposed to predominate over muscle hypertrophy during youth (Faigenbaum et al., 2013), although this varies across the 14–18-year age range and between sexes, and the mechanism of IMT may also operate differently in young people as a result. Therefore, studies conducted in young populations could provide specific data regarding the safe dosage, effective duration, and transfer of respiratory training to performance. However, researchers have reported limited evidence regarding the effects of IMT on comprehensive lung function parameters in young athletes (Shei et al., 2022).

On the other hand, plyometric training is a potential strength training modality that can indirectly improve respiratory function (Ramirez-Campillo et al., 2020a). Plyometric training represents a neuromuscular conditioning approach emphasizing the stretch-shortening cycle to enhance power output and neuromuscular efficiency (Ballerini et al., 2022). While primarily recognized for its effects on lower extremity power development, plyometric training may indirectly influence respiratory function through enhanced core stability, postural control, and overall cardiovascular conditioning (Ramirez-Campillo et al., 2020a). Plyometric training has been proposed to improve trunk musculature and intra-abdominal pressure dynamics and, in principle, could thereby influence the mechanical efficiency of the diaphragm and accessory respiratory muscles (Dias et al., 2017); however, this specific pathway is inferred from general core-training literature rather than established directly in respiratory-outcome studies, and conventional spirometric volumes are additionally strongly influenced by age, sex, height, and maturation. Furthermore, by increasing cardiovascular loading (Narayanan et al., 2025), plyometric training may stimulate ventilatory adaptations beyond those achieved through IMT alone. This mechanism may be crucial for adolescent athletes, where neuromuscular coordination, trunk stability, and the integration of the respiratory and postural subsystems are still developing. These references support the broader argument that respiratory, neuromuscular, and metabolic systems interact during athletic training, and are cited here as contextual evidence rather than as direct support for inspiratory-muscle-training- or pupillary-light-reflex-specific adaptations (Soslu et al., 2026; Devrilmez and Soslu, 2022).

While inspiratory muscle training reliably enhances respiratory muscle strength (typical maximal inspiratory pressure gains of 25–35 cmH_2_O in adult protocols of varying intensity and duration) (Ren et al., 2025), a range that may not directly generalize to youth athletes, its effects on conventional spirometric outcomes appear population-dependent, with swimmers showing isolated strength improvements whereas runners demonstrate concurrent gains in both respiratory muscle performance and lung function parameters (Carvajal-Tello et al., 2024).

Pulmonary Function Tests (PFT) and pupillary light reflex (PLR) are two potentially complementary biomarkers that may enable a broader assessment of training-induced physiological adaptations in young athletes (Giovannangeli et al., 2024; Okrzymowska et al., 2019), although the mechanistic relationship between pulmonary function and PLR-derived autonomic indices remains insufficiently established. PFT are a method that comprehensively assesses the mechanical and functional efficiency of the respiratory system. FEV_1_, FVC, and PEF reflect integrated pulmonary mechanics and expiratory effort rather than being direct measures of respiratory muscle strength; maximal inspiratory pressure (MIP) is more directly related to inspiratory muscle strength (Miller et al., 2005). These variables provide important information for assessing pulmonary adaptations associated with different types of physical training (Zugic et al., 2015). In addition, the PLR provides a non-invasive window into autonomic nervous system function, reflecting the balance between sympathetic and parasympathetic activity (Padmavathi et al., 2024). PLR parameters, including constriction velocity, maximum constriction percentage, and re-dilation velocity, have been validated as sensitive indicators of autonomic adaptation to training stress (Sivakumar et al., 2020). To the authors’ knowledge, no previous studies have investigated PLR responses to IMT or plyometric training interventions in youth athletes. The specificity of training adaptations suggests that IMT and plyometric training may elicit distinct physiological responses. While IMT directly targets respiratory muscle function, plyometric training may influence autonomic function through different pathways involving neuromuscular coordination and cardiovascular stress adaptation. Understanding these differential effects is crucial for developing evidence-based training prescriptions for youth middle-distance runners.

The primary objective of this study was to compare the effects of 12 weeks of IMT and plyometric training programs on lung function parameters and PLR characteristics in young middle-distance runners. The study problem statement was: Do 12 weeks of IMT and plyometric training programs produce significant differences in PFT and PLR parameters in young middle-distance runners? Based on the specificity-of-training principle, we hypothesized that: (1) IMT would produce greater improvements in FEV_1_ (the prespecified primary outcome) than plyometric training or control; (2) IMT would likewise produce greater gains in other spirometric parameters and MIP; (3) plyometric training would produce smaller, secondary gains in pulmonary parameters relative to IMT; and (4) IMT and plyometric training would be associated with differential, exploratory changes in individual PLR variables consistent with their respective primary physiological targets.

## 2 Materials and methods

### 2.1 Study design

This investigation was a three-arm, parallel-group, single-centre randomized controlled trial with pre- and post-intervention assessments at baseline and 12 weeks. The trial was conducted in accordance with the Declaration of Helsinki (World Medical A, 2013) and reported following the CONSORT 2010 guidelines for randomized trials (Schulz et al., 2010). The study protocol was approved by the Institutional Review Board (AU/1HEC/1/2023/32). This trial was not prospectively registered in a clinical trials registry. The full protocol and statistical analysis plan are available from the corresponding author upon reasonable request.

### 2.2 Power analysis and sample size calculation

Sample size calculation was based on anticipated changes in forced expiratory volume in FEV_1_ as the primary outcome measure. Based on previous IMT studies in athletic populations, we expected a moderate to large effect size (Cohen’s d = 0.65) for between-group differences in FEV_1_ improvement. Using G*Power 3.1.9.7 (Heinrich Heine University, Düsseldorf, Germany), we specified an F-test for a one-way, fixed-effects, omnibus ANOVA across the three groups, targeting the overall between-group difference in 12-week FEV_1_ change (α = 0.05, power [1-β] = 0.80), which indicated a minimum of 24 participants per group; the anticipated effect size was derived from FEV_1_ findings in athletic (not youth-specific) populations (Alnuman and Alshamasneh, 2022; Mackała et al., 2020). Anticipating a 20% dropout rate, we aimed to recruit 30 participants per group (total n = 90) (see **Figure 1**).

**Figure 1:**
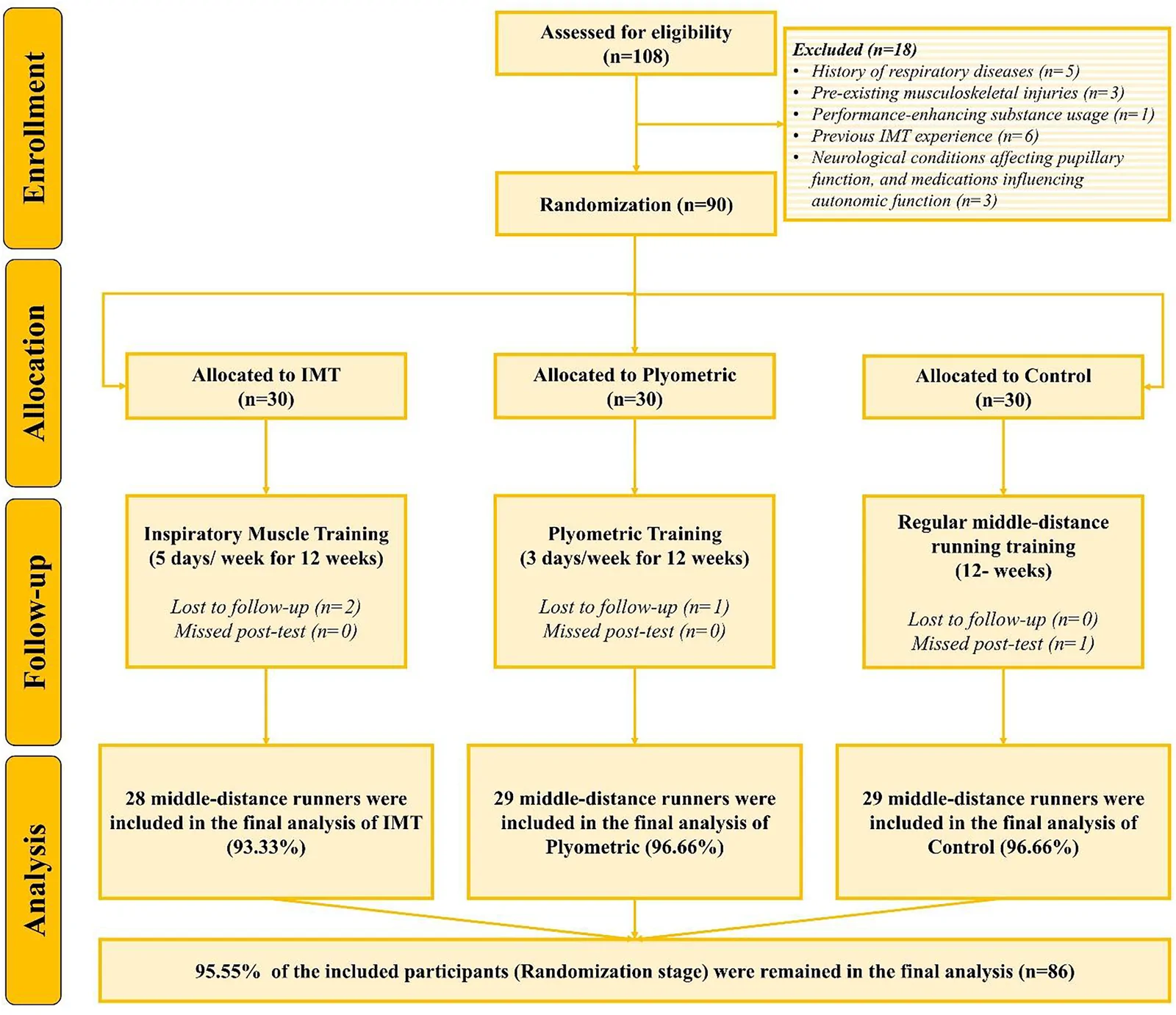
Consort flow diagram.

### 2.3 Participants

Ninety competitive youth middle-distance runners (800m-3000m events) aged 14–18 years were recruited from regional athletics clubs and schools in Tamil Nadu, India, between March and May 2024. Eligible participants were aged 14–18 years with minimum two years of structured middle-distance running experience and current training volume of at least four sessions per week. All participants required ability to complete baseline assessments and provided written parental consent with participant assent following ethical guidelines for minors (Bruzzese and Fisher, 2003).

Exclusion criteria included history of respiratory diseases (asthma, exercise-induced bronchoconstriction; COPD was listed as an exclusion criterion for completeness but was not anticipated in this adolescent cohort), current respiratory infections, musculoskeletal injuries limiting participation, performance-enhancing substance use, previous IMT experience, neurological conditions affecting pupillary function, and medications influencing autonomic function. These criteria minimized confounding variables and ensured participant safety throughout the intervention period (**Figure 1**).

All baseline and post-intervention assessments were conducted in the morning between 08:00 and 11:00 h to minimize circadian influences on pulmonary function and autonomic measures. General ambient conditions at the testing site (outside the temperature-controlled spirometry room and the darkened PLR testing room, described in §2.6–§2.7) were monitored using a digital environmental meter (Mastech MS830L+ Digital Multimeter, China) for descriptive reporting, with recorded values of 28–32 °C, relative humidity 55–65%, and wind speed 2–4 m/s. Participants were instructed to maintain usual hydration and to avoid caffeine, alcohol, and strenuous exercise for a minimum of 24 h prior to all testing (spirometry and PLR alike) to ensure measurement validity.

### 2.4 Randomization and blinding

Participants were randomly allocated to one of three intervention groups using computer-generated randomization with block sizes of 6. Allocation concealment was ensured through sequentially numbered, sealed, opaque envelopes, as recommended to prevent selection bias and maintain methodological rigor (Sil et al., 2019). Due to the nature of the interventions, participants and trainers could not be blinded to group assignment. However, outcome assessors remained blinded to group allocation throughout the study period, adhering to best practices to minimize performance and detection biases in randomized controlled trials (Chess and Gagnier, 2013).

### 2.5 Interventions

#### 2.5.1 Inspiratory muscle training group

The IMT intervention utilized threshold inspiratory muscle trainers (Threshold IMT, Philips Respironics, USA), which provide consistent resistance throughout the inspiratory range of motion and have been validated for respiratory muscle strengthening in athletic populations (Geddes et al., 2005). Participants performed IMT sessions five days per week for 12 weeks, completing 30 inspiratory efforts per session conducted twice daily (morning and evening; 300 total inspiratory efforts per week) to optimize training stimulus and minimize respiratory muscle fatigue (Romer and McConnell, 2003). The training intensity was initially set at 30% of maximum inspiratory pressure (MIP) in week 1 and progressed by 5 percentage points each week (reaching 75% MIP by week 10 and 80% MIP by week 11), maintaining 80% MIP through week 12, consistent with progressive overload principles for respiratory muscle adaptation (HajGhanbari et al., 2013). Weekly MIP assessments were conducted to adjust training loads and ensure appropriate progression throughout the intervention period; we acknowledge that this repeated maximal respiratory effort may itself have constituted an additional training stimulus, which is noted as a limitation. The first two weeks of training were directly supervised to ensure proper technique and breathing patterns, with subsequent sessions conducted independently at home with weekly check-ins (adherence logs and verbal report) to monitor adherence and address any technical concerns (Illi et al., 2012); objective device-based monitoring of home sessions was not used, which is acknowledged as a limitation, as is the resulting difference in supervision intensity relative to the fully supervised plyometric-training sessions (§2.5.2).

#### 2.5.2 Plyometric training group

The plyometric training intervention followed established guidelines for youth athletes, emphasizing progressive development of neuromuscular power and reactive strength capabilities (Lloyd et al., 2022). Training sessions were conducted three times per week for 12 weeks, with each session lasting 45–60 minutes including warm-up, plyometric exercises, and cool-down phases. The exercise selection included depth jumps, box jumps, horizontal bounds, reactive jumps, and medicine ball throws, targeting multiple movement planes and athletic movement patterns relevant to middle-distance running performance (Ramirez-Campillo et al., 2020b). Training volume ranged from 80–120 foot contacts per session (240–360 total weekly contacts at three sessions per week), progressing systematically from low to high intensity movements to ensure appropriate neuromuscular adaptation while minimizing injury risk, consistent with youth training-load recommendations (Markovic and Mikulic, 2010). Exercise intensity progression followed a systematic approach, beginning with bilateral movements and advancing to unilateral exercises, while concurrently increasing drop heights and reactive components to enhance stretch-shortening cycle efficiency. This frequency of three sessions per week was chosen to allow sufficient stimulus for adaptation while providing adequate recovery, consistent with recommendations for youth athletes to optimize performance gains and minimize overuse injuries (Lloyd et al., 2022). A detailed week-by-week progression table (exercise selection, sets/repetitions, contact counts, box/drop heights, rest intervals, and progression criteria) is available from the corresponding author upon request. Participants were instructed to maintain their usual dietary habits and refrain from additional respiratory or plyometric training outside the study; all three groups, including the IMT and plyometric groups, continued their regular middle-distance running training throughout the study, with the assigned intervention delivered in addition to usual running training. Total running training load was not independently quantified across groups beyond coach-confirmed adherence to normal routines. All sessions were directly supervised by certified strength and conditioning specialists (NSCA-CSCS; National Strength and Conditioning Association, USA) to ensure correct technique execution, appropriate progression, and immediate feedback on movement quality; this full on-site supervision contrasts with the largely home-based IMT protocol (§2.5.1), a difference in researcher contact that may have differentially influenced expectation and motivation between groups.

#### 2.5.3 Control group

Control group participants maintained their regular middle-distance training programs throughout the 12-week study period without receiving additional interventions, allowing for assessment of natural training adaptations and seasonal variation effects. Detailed training logs were completed weekly to monitor training consistency, volume, and intensity, ensuring that any observed differences could be attributed to the specific interventions rather than variations in baseline training exposure. Regular communication with coaches (Diploma in Sports Coaching-certified) confirmed adherence to normal training routines and identified any significant changes in training patterns that might influence outcome measures.

### 2.6 Primary outcome measurements

#### 2.6.1 Pulmonary function testing

Comprehensive pulmonary function assessment was conducted using a calibrated digital spirometer (Helio, 407, RMS, India) following American Thoracic Society/European Respiratory Society standardized protocols (Miller et al., 2005), updated per the current ERS/ATS technical standard on interpretive strategies (Stanojevic et al., 2022), to ensure measurement accuracy and reproducibility. Testing was performed in a temperature-controlled environment (20–22 °C) with participants in a seated position following the 24-hour caffeine/alcohol/strenuous-exercise abstention described in §2.3 and a 30-minute pre-test rest period to minimize confounding variables. Each participant performed a minimum of three acceptable maneuvers meeting ATS/ERS acceptability and repeatability criteria (<150 mL variation between the two largest FEV_1_ and FVC values); the largest acceptable FEV_1_ and FVC values were used for analysis, per standard spirometry practice. Primary spirometry parameters included forced expiratory volume in FEV_1_, FVC, FEV_1_/FVC ratio, and PEF, representing comprehensive assessment of lung function and airway dynamics. Additionally, maximum inspiratory pressure (MIP) was measured using a digital manometer (MicroRPM, CareFusion, USA) to assess inspiratory muscle strength, with participants performing maximum inspiratory efforts against an occluded airway from residual volume (Parreira et al., 2007). Maximum expiratory pressure (MEP) was not measured in this study.

### 2.7 Secondary outcome measurements

#### 2.7.1 Pupillary light reflex assessment

Pupillary light reflex measurements were conducted using a portable dynamic pupillometer in a standardized dark room environment to eliminate external light contamination and ensure consistent testing conditions (Maruthy et al., 2020). Prior to testing, participants underwent a five-minute dark adaptation period while seated comfortably to allow pupillary stabilization and optimize baseline measurements. The testing protocol involved presentation of a standardized light stimulus (180 cd/m^2^) for 3.2 seconds to the right eye only, with both eyes left open and pupil diameter recorded simultaneously from both eyes at 30 Hz a sampling rate consistent with prior validated pupillometry protocols (Kumar et al., 2019; Siva Kumar et al., 2022) to capture the direct response in the stimulated (right) eye and the consensual response in the unstimulated (left) eye. PLR parameters analyzed included initial pupil diameter (mm); peak constriction velocity (mm/s), defined as the maximum rate of pupillary constriction during the constriction phase; maximum constriction percentage, calculated as the relative decrease from baseline diameter; and peak re-dilation velocity (mm/s), defined as the maximum rate of pupillary diameter increase during the recovery phase (Kumar et al., 2019). Time to maximum constriction was not analyzed in the present study. All measurements were performed at consistent times of day (08:00-11:00 hours) to minimize circadian influences on autonomic function, and participants were instructed to avoid alcohol, caffeine, and strenuous exercise for 24 hours prior to testing to ensure measurement validity.

### 2.8 Statistical analysis

Data analysis was performed using SPSS version 28.0 (IBM Corp., Armonk, NY). Normality was assessed using Shapiro-Wilk tests and visual inspection of Q-Q plots. Baseline characteristics were descriptively compared across groups (one-way ANOVA for continuous variables, chi-square tests for categorical variables); baseline significance testing is presented descriptively only, consistent with the view that it is not required to establish balance in a randomized trial (Winston et al., 2020). Because four randomized participants withdrew before outcome assessment, the primary analysis was a complete-case analysis of the 86 participants (86/90, 95.6%) who completed baseline and 12-week assessments; no imputation was performed for missing data, which is discussed as a limitation relative to a formal intention-to-treat approach. For each outcome, between-group differences at 12 weeks were tested using ANCOVA, with baseline value and sex included as prespecified covariates given their known associations with pulmonary function and PLR parameters; the omnibus group-effect p-value from each ANCOVA model is reported as the between-group p-value in **Tables 1** and **2**. Within-group baseline-to-12-week changes were additionally tested using paired t-tests, with Bonferroni-corrected significance thresholds applied within each outcome domain (pulmonary function: FEV_1_, FVC, FEV_1_/FVC, PEF, MIP; PLR: initial diameter, constriction velocity, maximum constriction, re-dilation velocity). Standardized within-group effect sizes were calculated using Cohen’s d with 95% confidence intervals and classified as trivial (0–0.20), small (0.21–0.60), moderate (0.61–1.20), large (1.21–2.00), or very large (2.01–4.00) (Hopkins et al., 2009); partial eta-squared would also be an appropriate complementary omnibus effect-size metric for the ANCOVA models. Because FEV_1_ alone was used for the sample-size calculation, findings for the remaining pulmonary and all PLR parameters should be regarded as hypothesis-generating. Statistical significance was set at *p* < 0.05.

**Table 1:** Effects of ınterventions on pulmonary function parameters*.

Parameter	Group	Baseline mean (SD)	Post-intervention mean (SD)	Within-group change from baseline (95% CI) ^a^	Within-group effect size (95% CI) ^b^	Between-group *p*-value ^c^
FEV_1_ (L)	IMT	4.12 (0.23)	4.45 (0.25)	0.33 (0.28 to 0.38) ^d^	1.35 (0.91 to 1.79)	<0.001
	Plyometric	4.08 (0.26)	4.25 (0.28)	0.17 (0.12 to 0.22) ^e^	0.63 (0.25 to 1.01)	
	Control	4.10 (0.24)	4.13 (0.26)	0.03 (−0.02 to 0.08)	0.12 (−0.24 to 0.48)	
FVC (L)	IMT	4.89 (0.31)	5.28 (0.33)	0.39 (0.33 to 0.45) ^d^	1.23 (0.80 to 1.66)	<0.001
	Plyometric	4.85 (0.29)	5.02 (0.32)	0.17 (0.11 to 0.23)	0.56 (0.19 to 0.93)	
	Control	4.87 (0.30)	4.91 (0.31)	0.04 (−0.02 to 0.10)	0.13 (−0.23 to 0.49)	
FEV_1_/FVC	IMT	0.84 (0.04)	0.84 (0.04)	0.00 (−0.01 to 0.01)	0.00 (−0.36 to 0.36)	0.52
	Plyometric	0.84 (0.05)	0.85 (0.04)	0.01 (0.00 to 0.02)	0.22 (−0.14 to 0.58)	
	Control	0.84 (0.04)	0.84 (0.04)	0.00 (−0.01 to 0.01)	0.00 (−0.36 to 0.36)	
PEF (L/min)	IMT	628 (45)	672 (48)	44 (37 to 51) ^d^	0.95 (0.54 to 1.36)	<0.001
	Plyometric	624 (47)	641 (49)	17 (10 to 24)	0.35 (−0.01 to 0.71)	
	Control	626 (46)	631 (47)	5 (−2 to 12)	0.11 (−0.25 to 0.47)	
MIP (cmH_2_O)	IMT	118 (16)	156 (18)	38 (34 to 42) ^d^	2.25 (1.71 to 2.79)	<0.001
	Plyometric	116 (17)	128 (19)	12 (8 to 16) ^e^	0.67 (0.29 to 1.05)	
	Control	117 (15)	119 (16)	2 (−2 to 6)	0.13 (−0.23 to 0.49)	

FEV_1_, forced expiratory volume in 1 second; FVC, forced vital capacity; IMT, inspiratory muscle training; MIP, maximal inspiratory pressure; PEF, peak expiratory flow.a: Raw within-group mean change from baseline to 12 weeks (unadjusted paired difference); b: Cohen d calculated as within-group change divided by pooled SD; c: omnibus between-group p-value from ANCOVA of 12-week values, adjusted for baseline value and sex; d: p<0.001 for comparison with baseline (paired t test with Bonferroni correction); e: p < 0.05 for comparison with baseline (paired t test with Bonferroni correction).

**Table 2:** Effects of ınterventions on pupillary light reflex parameters.

Parameter	Group	Baseline mean (SD)	Post-intervention mean (SD)	Within-group change from baseline (95% CI) ^a^	Within-group effect size (95% CI) ^b^	Between-group *p*-value ^c^
Initial Diameter (mm)	IMT	6.8 (0.9)	6.9 (0.8)	0.1 (−0.1 to 0.3)	0.12 (−0.24 to 0.48)	0.23
	Plyometric	6.7 (0.8)	6.8 (0.9)	0.1 (−0.1 to 0.3)	0.11 (−0.25 to 0.47)	
	Control	6.8 (0.9)	6.7 (0.8)	−0.1 (−0.3 to 0.1)	−0.12 (−0.48 to 0.24)	
Constriction Velocity (mm/s)	IMT	5.2 (0.8)	6.1 (0.9)	0.9 (0.7 to 1.1) ^d^	1.08 (0.66 to 1.50)	0.003
	Plyometric	5.1 (0.9)	5.4 (0.8)	0.3 (0.1 to 0.5)	0.36 (−0.00 to 0.72)	
	Control	5.2 (0.8)	5.1 (0.9)	−0.1 (−0.3 to 0.1)	−0.12 (−0.48 to 0.24)	
Maximal Constriction (%)	IMT	32.4 (4.2)	35.8 (4.6)	3.4 (2.1 to 4.7) ^e^	0.77 (0.37 to 1.17)	0.02
	Plyometric	32.1 (4.5)	33.9 (4.8)	1.8 (0.5 to 3.1)	0.38 (0.02 to 0.74)	
	Control	32.3 (4.3)	32.0 (4.4)	−0.3 (−1.6 to 1.0)	−0.07 (−0.43 to 0.29)	
Re-dilation Velocity (mm/s)	IMT	3.2 (0.7)	3.6 (0.8)	0.4 (0.2 to 0.6) ^e^	0.53 (0.16 to 0.90)	0.01
	Plyometric	3.1 (0.6)	3.8 (0.7)	0.7 (0.5 to 0.9) ^e^	1.06 (0.64 to 1.48)	
	Control	3.2 (0.7)	3.1 (0.6)	−0.1 (−0.3 to 0.1)	−0.15 (−0.51 to 0.21)	

IMT, inspiratory muscle training; a: Raw within-group mean change from baseline to 12 weeks (unadjusted paired difference); b:Cohen d calculated as within-group change divided by pooled SD; c: omnibus between-group p-value from ANCOVA of 12-week values, adjusted for baseline value and sex; d: p < 0.01 for comparison with baseline (paired t test with Bonferroni correction); e: p < 0.05 for comparison with baseline (paired t test with Bonferroni correction).

## 3 Results

### 3.1 Adherence to intervention

In this study 108 adolescent athletes were assessed for eligibility. Of these, 18 were excluded (**Figure 1**). Ninety participants were randomly assigned to inspiratory muscle training (IMT; n=30), plyometric training (n=30), or control (n=30) groups. Four participants withdrew during the intervention (2 from IMT, 1 from plyometric, 1 from control), leaving 86 participants (95.6% retention) who completed the study and were included in the complete-case primary analysis; the four withdrawn participants were not included via imputation.

Baseline characteristics were well balanced across groups (**Table 3**). The mean age was 16.0 years (SD 1.4), mean height 168.4 cm (SD 8.8), and 62.2% were male. Participants had been engaged in athletic training for a mean of 4.1 years (SD 1.7). No significant differences were observed between groups for any baseline characteristic (all *p*>0.05).

**Table 3:** Baseline characteristics of study participants.

Characteristic	IMT (n=30) mean (SD)	Plyometric training (n=30) mean (SD)	Control group (n=30) mean (SD)	*p*-value^a^
Demographics				
Age (years)	16.2 (1.4)	15.9 (1.3)	16.0 (1.5)	0.67
Male sex, No. (%)	18 (60.0)	17 (56.7)	19 (63.3)	0.85
Anthropometrics				
Height (cm)	168.4 (8.7)	167.8 (9.2)	169.1 (8.4)	0.82
Weight (kg)	58.7 (7.3)	59.2 (8.1)	57.9 (7.8)	0.74
BMI (kg/m^2^)	20.7 (2.1)	21.0 (2.3)	20.3 (2.0)	0.46
Athletic Background				
Training experience (years)	4.2 (1.6)	4.0 (1.8)	4.1 (1.7)	0.89

BMI, body mass index; IMT, inspiratory muscle training. a: P-values calculated using one-way ANOVA for continuous variables and χ^2^ test for categorical variables.

Training adherence was high in both intervention groups: 94.2% (SD 5.8%) in the IMT group and 91.7% (SD 6.4%) in the plyometric group. No serious adverse events were reported during the study period.

### 3.2 Pulmonary function test results

#### 3.2.1 Between-group comparisons

Significant between-group differences at 12 weeks (ANCOVA, adjusted for baseline value and sex) were observed for forced expiratory volume in FEV_1_, FVC, PEF, and maximal inspiratory pressure (MIP) (all p < 0.001), but not for FEV_1_/FVC ratio (p = 0.52) (see **Table 1**). These adjusted between-group comparisons, rather than the within-group changes reported in §3.2.2, constitute the primary evidence for intervention effects in this randomized trial.

At 12 weeks, the IMT group demonstrated significantly greater improvements in FEV_1_ compared with control (mean difference = 0.30 L, 95% CI = 0.22 to 0.38, *p* < 0.001, Cohen d = 1.35) and plyometric groups (mean difference = 0.16 L, 95% CI = 0.08 to 0.24, *p* < 0.001). Similar patterns were observed for FVC (IMT vs control: mean difference = 0.35 L, 95% CI = 0.25 to 0.45, *p* < 0.001, Cohen d = 1.23).

The most pronounced effect was observed in MIP, with the IMT group showing a mean increase of 38 cmH_2_O (95% CI = 34 to 42, *p* < 0.001, Cohen d = 2.25) compared with 12 cmH_2_O in the plyometric group (95% CI = 9 to 15, *p* < 0.001, Cohen d = 0.67) and 2 cmH_2_O in controls (95% CI = 0 to 4; *p* = 0.06). The magnitude of the effects for the pulmonary function parameters ranged from trivial to very large, as illustrated in **Figure 2**.

**Figure 2:**
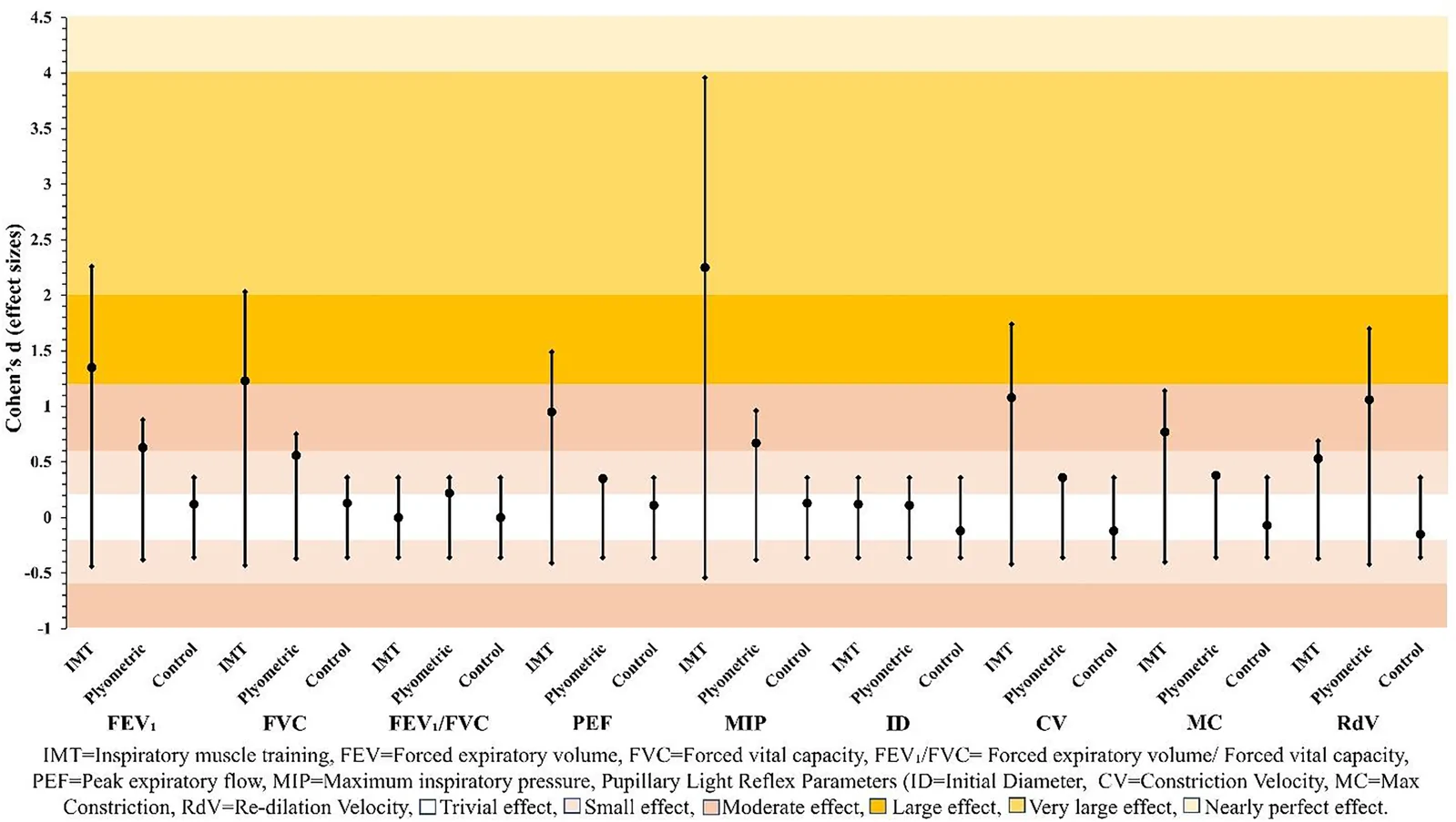
Magnitude of change from baseline to post-intervention in pulmonary function and pupillary light reflex parameters.

#### 3.2.2 Within-group changes

As secondary, descriptive context to the adjusted between-group comparisons above, within-group changes from baseline are summarized here. In the IMT group, statistically significant improvements from baseline were observed for all pulmonary parameters except FEV_1_/FVC: FEV_1_ increased by 8.0% (95% CI = 6.1% to 9.9%, *p* < 0.001), FVC by 8.0% (95% CI = 5.9% to 10.1%, *p* < 0.001), PEF by 7.0% (95% CI = 5.3% to 8.7%, *p* < 0.001), and MIP by 32.2% (95% CI = 28.4% 36.0%, *p* < 0.001).

The plyometric group showed modest but significant improvements in FEV_1_ (4.2%; 95% CI, 2.5%-5.9%; *p* = 0.02) and MIP (10.3%; 95% CI, 7.8%-12.8%; *p* = 0.01). No significant within-group changes were observed in the control group for any pulmonary parameter.

### 3.3 Secondary outcomes: pupillary light reflex

#### 3.3.1 Between-group comparisons

Significant between-group differences at 12 weeks (ANCOVA, adjusted for baseline value and sex) were observed for pupillary constriction velocity (p = 0.003), maximal constriction percentage (p = 0.02), and re-dilation velocity (p = 0.01), but not for initial pupil diameter (p = 0.23) (see **Table 2**). As with the pulmonary outcomes, these adjusted between-group comparisons are the primary evidence; within-group changes are reported in §3.3.2 as secondary, descriptive context.

The IMT group demonstrated significantly greater improvement in constriction velocity compared with control (mean difference = 1.0 mm/s, 95% CI = 0.7 to 1.3, *p* < 0.001, Cohen d = 1.08). The plyometric group showed the largest within-group improvement in re-dilation velocity (mean increase = 0.7 mm/s, 95% CI = 0.6 to 0.8, p < 0.001, Cohen d = 1.06); this within-group comparison is descriptive only, as direct pairwise adjusted contrasts between the plyometric and IMT groups for re-dilation velocity are not reported. The magnitude of the effects for the Pupillary Light Reflex Parameters ranged from trivial to very large, as illustrated in **Figure 2**.

#### 3.3.2 Within-group changes

The IMT group showed significant improvements in constriction velocity with 17.3% increase (95% CI = 13.2% to 21.4%, *p* = 0.002), maximal constriction with a 10.5% increase (95% CI = 7.1% to 13.9%, p = 0.04), and re-dilation velocity with 12.5% increase (95% CI = 8.3% to 16.7%, *p* = 0.03).

In the plyometric group, significant improvement was observed only in re-dilation velocity with 22.6% increase (95% CI = 19.1% to 26.1%, *p* = 0.01). No significant changes were observed in the control group for any pupillary parameter.

### 3.4 *Post hoc* analyses

Exploratory correlation analyses revealed moderate positive associations between changes in MIP and changes in pupillary constriction velocity (*r* = 0.42, 95% CI = 0.24 to 0.57, *p* < 0.001) and between changes in FEV_1_ and changes in maximal constriction percentage (*r* = 0.38, 95% CI = 0.19 to 0.54, *p* = 0.002). These correlations were computed on change scores pooled across all three groups and persisted after adjustment for age, sex, baseline training experience, and intervention group; given the pooled design, these exploratory correlations should be interpreted cautiously, as they may partly reflect between-group separation rather than purely within-participant physiological coupling, and are additionally subject to regression-to-the-mean when computed on change scores.

## 4 Discussion

To the authors’ knowledge, this study represents the first randomized controlled trial to directly compare the effects of inspiratory muscle training and plyometric conditioning on both pulmonary function and autonomic nervous system markers in youth middle-distance runners. The principal findings demonstrate that while IMT produced superior improvements in spirometry parameters, both interventions elicited distinct autonomic adaptations as evidenced by differential pupillary light reflex responses. These findings extend current understanding by demonstrating training-specific differences in PLR indices; whether these differences reflect generalized “autonomic signatures” with implications for athletic preparation in youth populations requires further investigation, as PLR is an indirect autonomic index influenced by multiple non-training factors.

The IMT group demonstrated significant improvements across multiple spirometry parameters, with effect sizes ranging from moderate to very large (d = 0.95–2.25; PEF 0.95, FVC 1.23, FEV_1_ 1.35, MIP 2.25). The 8.0% improvement in FEV_1_ and 8.0% increase in FVC align with recent findings in athletic populations. The magnitude of these improvements is clinically meaningful, as FEV_1_ changes exceeding 5% are considered physiologically significant in athletic populations and may translate to enhanced ventilatory capacity during high-intensity exercise. Notably, the 32.2% improvement in maximum inspiratory pressure confirms the specificity of IMT adaptations, consistent with the overload principle applied to respiratory muscles.

These findings are corroborated by Carvajal-Tello et al. (2024), whose meta-analysis of swimmers using IMT demonstrated a significant pooled improvement in MIP (MD = 29.35 cmH_2_O, p < 0.01), while FEV_1_ and FVC remained unchanged (Carvajal-Tello et al., 2024). The discrepancy between our findings (improved FEV_1_/FVC) and those of Carvajal-Tello et al. (2024) may reflect sport-specific differences in baseline respiratory function, as swimmers often demonstrate superior lung volumes due to chronic training adaptations to aquatic environments. Our youth middle-distance runners, in contrast, may have had greater capacity for improvement in conventional spirometry parameters. Similarly, Ren et al. (2025) reported that 8 weeks of IMT in amateur runners significantly improved both MIP and MEP (p < 0.01), alongside enhanced exercise tolerance as evidenced by increased time to exhaustion and reduced blood lactate accumulation (Ren et al., 2025). The shorter duration in Ren et al.’s study (8 weeks) compared to our 12-week protocol raises the possibility that respiratory muscle strength adaptations may occur relatively early in a training block; however, our study did not include an interim assessment and therefore cannot confirm the time-course of adaptation within our own cohort, so this cross-study comparison should be regarded as speculative.

The magnitude of respiratory muscle strength improvements observed in our youth runners is consistent with dose-response relationships established in adult populations. Ren et al. (2025) demonstrated that high-intensity IMT protocols produced superior improvements in exercise tolerance compared to low-intensity protocols, suggesting that the progressive loading approach employed in our study may have optimized adaptation (Ren et al., 2025). Furthermore, Fernández-Lázaro et al. (2025) reported modest but significant improvements in maximum voluntary ventilation and MIP following 12 weeks of training with an elevation training mask in post-COVID CrossFit practitioners, reinforcing the efficacy of respiratory resistance training across diverse populations and training contexts (Fernández-Lázaro et al., 2025). The post-COVID population studied by Fernández-Lázaro et al. (2025) likely experienced respiratory deconditioning, potentially explaining their more modest improvements compared to our healthy youth athletes. This suggests that IMT protocols may require adjustment based on baseline respiratory function and training status.

The plyometric group showed modest but significant improvements in FEV_1_ (4.2%) and MIP (10.3%), suggesting that neuromuscular training may provide secondary benefits to respiratory function. These secondary respiratory adaptations may result from several mechanisms: (1) enhanced core muscle strength improving trunk stability during forced expiration, (2) increased intrathoracic pressure generation capacity due to improved abdominal muscle function during landing phases, and (3) potential cross-transfer effects from explosive lower-limb contractions requiring coordinated respiratory-locomotor coupling. These three proposed mechanisms are speculative and were not directly tested in this study.

This finding supports the concept that enhanced core stability and postural control, common adaptations to plyometric training, may optimize respiratory mechanics during dynamic activities. The 12-week duration employed in our study aligns with recommendations from recent meta-analyses. A recent systematic review and meta-analysis demonstrated that plyometric-based training significantly improved running economy in recreational runners, particularly at speeds of 14 km/h (SMD = -0.42, p = 0.05), with optimal adaptations occurring following 6–12 weeks of progressive training at 2–3 sessions per week (Deviatika and Rayhan, 2025). Similarly, Kryeziu et al. (2023) reported significant improvements in speed and explosive strength indicators following a 12-week plyometric program in adolescents (p < 0.05), suggesting that the duration of our intervention was sufficient to elicit meaningful adaptations (Kryeziu et al., 2023). The alignment of our findings with these meta-analytic recommendations strengthens confidence in the external validity of our 12-week periodized approach for youth athletes.

Interestingly, Eihara et al. (2022) found that heavy resistance training produced greater improvements in running economy compared to plyometric training in long-distance runners, with effect sizes of g = -0.32 versus g = -0.13, respectively. This suggests that while plyometric training offers benefits, its effects on physiological parameters may be more modest than other resistance-based modalities (Eihara et al., 2022). However, the neuromuscular and coordinative benefits of plyometric training extend beyond simple respiratory improvements, potentially contributing to the distinct autonomic adaptations observed in our study (Deviatika and Rayhan, 2025). Future research should examine whether combined plyometric-resistance protocols produce additive or synergistic effects on both respiratory and neuromuscular adaptations in youth runners.

The preservation of the FEV_1_/FVC ratio across all groups indicates that improvements occurred without compromising airway function, an important safety consideration for youth athletes. The lack of change in this ratio suggests that adaptations were primarily related to enhanced respiratory muscle strength rather than airway remodelling. This is reassuring given concerns about exercise-induced bronchoconstriction in youth athletes, as it indicates that neither IMT nor plyometric training adversely affected airway caliber or induced pathological airway changes (Carvajal-Tello et al., 2024).

The pupillary light reflex findings provide novel insights into training-specific autonomic adaptations (Winston et al., 2020). The IMT group’s within-group improvement in constriction velocity (17.3% increase) may reflect enhanced parasympathetic responsiveness, potentially related to improved respiratory-cardiovascular coupling through strengthened respiratory muscles (Gajardo et al., 2019; Sivakumar et al., 2020). A possible, unmeasured mechanistic pathway may involve: (1) strengthened respiratory muscles reducing the work of breathing and associated sympathetic activation during exercise, (2) improved ventilatory efficiency enhancing baroreflex sensitivity through more stable intrathoracic pressure oscillations, and (3) possible vagal training effects from repetitive inspiratory resistance loading activating mechanoreceptors in the respiratory muscles and chest wall. These adaptations manifest as improved heart rate variability and enhanced post-exercise recovery, though these outcomes were not measured in the current study.

Conversely, the plyometric group demonstrated a within-group improvement in re-dilation velocity (22.6% increase); re-dilation velocity has been associated with sympathetic nervous system function, although it is also influenced by parasympathetic withdrawal and measurement conditions, and should not be interpreted as an isolated marker of sympathetic activity. This finding aligns with the high-intensity, power-based nature of plyometric training, which may enhance sympathetic responsiveness through repeated exposure to rapid force development demands (Hung et al., 2025; Ramirez-Campillo et al., 2020a). The enhanced sympathetic reactivity may represent an adaptive response to the repeated high-intensity stretch-shortening cycles characteristic of plyometric exercise, which require rapid sympathetic activation for force production and metabolic mobilization. Whether this adaptation translates to improved reactive capacity during competitive racing scenarios requiring sudden pace changes or sprint finishes is speculative, as no running-performance outcome was measured in this study.

These differential autonomic adaptations suggest that training modality specificity extends beyond the primary target systems to influence broader physiological networks (Balagué et al., 2020). The divergent within-group PLR profiles observed with IMT versus plyometric training generate a testable hypothesis—namely, that periodized programs strategically combining both modalities might optimize autonomic balance in youth athletes—which was not tested in the present three-arm design and should be evaluated in future trials incorporating a combined-intervention arm before any practical recommendation (e.g., prioritizing IMT during high-volume phases and plyometric training during competitive periods) is made. The clinical relevance of these PLR changes requires further investigation, but they may represent early markers of training adaptation preceding performance improvements. Future research should examine whether PLR changes correlate with traditional performance markers (e.g., time trial performance, lactate threshold, running economy) and whether they predict training responsiveness or overtraining risk.

The superior respiratory adaptations to IMT versus plyometric training support the principle of training specificity, where adaptations are greatest in systems directly targeted by the intervention. These physiological findings suggest that IMT may offer greater benefit for pulmonary function outcomes than plyometric training in this population; however, as running performance, competition outcomes, and long-term effects were not assessed, we stop short of a prescriptive recommendation that IMT “should be prioritized.” The within-group 8% improvements in FEV_1_ and FVC observed with IMT exceed the typical 2–3% year-over-year improvements attributable to natural growth and maturation in adolescents, which is consistent with (though not definitive proof of) a genuine training-induced adaptation beyond normal maturation.

The moderate within-group improvements observed in the plyometric group raise the hypothesis of potential synergistic effects when combining neuromuscular and respiratory training approaches, a hypothesis not tested by the present three-arm design. Both Eihara et al. (2022) and Deviatika and Rayhan (2025) emphasize that longer training periods (10–14 weeks for resistance training; 6–12 weeks for plyometric training) produce greater effects, supporting the use of periodized, progressive training protocols that integrate multiple training modalities throughout a competitive season (Deviatika and Rayhan, 2025; Eihara et al., 2022). A practical periodization approach might involve: (1) 4–6 weeks of foundational IMT during the general preparation phase to establish respiratory muscle strength, (2) concurrent IMT and plyometric training during the specific preparation phase to develop both aerobic and neuromuscular capacities, and (3) maintenance-level IMT (2 sessions/week) during competitive periods while emphasizing plyometric and speed work.

The distinct autonomic signatures of each training modality further support this integrative approach. Coaches leverage IMT’s parasympathetic-enhancing effects during high-volume training blocks to facilitate recovery, while utilizing plyometric training’s sympathetic-activating properties during pre-competition tapering to maintain neural responsiveness despite reduced training volume. The high adherence rates (>90%) in both intervention groups indicate that these protocols are feasible and well-tolerated by youth athletes. The absence of adverse events supports the safety of progressive IMT and plyometric training in this population when appropriately supervised, consistent with the findings of Fernández-Lázaro et al. (2025) in post-infection athletes and Kryeziu et al. (2023) in adolescents (Fernández-Lázaro et al., 2025; Kryeziu et al., 2023).

### 4.1 Limitations

Several limitations should be acknowledged. First, the study population was limited to middle-distance runners from a specific geographic region, potentially limiting generalizability to other youth athletic populations or events. Second, the 12-week intervention period, while showing significant adaptations, may not capture longer-term training effects or determine optimal training durations. Third, the inability to blind participants to their intervention assignment may have influenced motivation and effort; participants and trainers were not blinded, no attention-matched or sham-IMT control was used, and blinding success among outcome assessors was not formally evaluated. Fourth, this trial was not prospectively registered in a clinical trials registry. Fifth, the primary analysis was a complete-case analysis (86/90 randomized participants, 95.6%) rather than a formal intention-to-treat analysis with imputation; the four withdrawn participants (2 IMT, 1 plyometric, 1 control) were excluded rather than imputed. Sixth, only FEV_1_ was incorporated into the sample-size calculation, so findings for the remaining pulmonary parameters and all PLR parameters should be regarded as hypothesis-generating despite the Bonferroni-corrected within-domain testing applied. Seventh, weekly MIP testing may itself have constituted an additional respiratory-muscle stimulus, home-based IMT sessions (from week 3 onward) were monitored only through adherence logs and weekly check-ins rather than objective device data, and the plyometric group received full on-site supervision while the IMT group did not—differences in researcher contact and monitoring intensity that may have differentially influenced adherence, motivation, and expectation between groups. Eighth, several factors known to influence PLR (recent screen exposure, sleep duration, psychological stress, time since last meal, menstrual-cycle phase, refractive correction, pre-adaptation ambient illumination, and recent medication or eye-drop use) were not systematically documented beyond the standardized testing window and pre-testing abstention instructions, and test–retest reliability of the PLR protocol was not separately assessed in this cohort. Ninth, the sample combined runners aged 14–18 years without a formal pubertal-maturation assessment (e.g., Tanner staging), so chronological age may not fully capture maturation-related variance across this range. Tenth, exploratory MIP–PLR and FEV_1_–PLR correlations were computed on change scores pooled across all three groups and, despite adjustment for group, may partly reflect between-group separation and are subject to regression-to-the-mean. Finally, the study did not include performance measures such as time trial results, limiting the ability to translate physiological improvements to competitive outcomes.

### 4.2 Future research directions

Future studies should investigate the dose-response relationships for both IMT and plyometric training in youth athletes, including optimal training frequencies, intensities, and durations. Additionally, research examining the combination of these interventions may reveal synergistic effects on both respiratory and neuromuscular systems. Longitudinal studies tracking athletes through multiple competitive seasons could provide insights into the long-term benefits and optimal timing of these interventions within annual training plans. Finally, investigating the relationship between PLR adaptations and actual performance improvements would enhance the clinical relevance of these autonomic markers.

## 5 Conclusion

In this complete-case analysis, inspiratory muscle training was associated with greater adjusted improvements in pulmonary function parameters than plyometric training in youth middle-distance runners. Both interventions were additionally associated with within-group changes in pupillary light reflex indices, consistent with, but not conclusive proof of, training-specific autonomic effects extending beyond the primary target systems; PLR is an indirect, multiply-determined marker, and these findings warrant confirmation with more comprehensive autonomic measures (e.g., heart-rate variability). These physiological findings may inform, but do not by themselves justify, prescriptive training recommendations for coaches and practitioners, given the absence of running-performance, competition, and long-term outcome data in the present trial.

## Data Availability

The raw data supporting the conclusions of this article will be made available by the authors, without undue reservation.
